# Hyperparameter Optimization Techniques for Designing Software Sensors Based on Artificial Neural Networks

**DOI:** 10.3390/s21248435

**Published:** 2021-12-17

**Authors:** Sebastian Blume, Tim Benedens, Dieter Schramm

**Affiliations:** Department of Mechanical and Process Engineering, Institute for Mechatronics and System Dynamics, University of Duisburg-Essen, 47057 Duisburg, Germany; Sebastian.Blume@uni-due.de (S.B.); Tim.Benedens@uni-due.de (T.B.)

**Keywords:** artificial neural networks, hyperparameter optimization, software sensors, intelligent transportation

## Abstract

Software sensors are playing an increasingly important role in current vehicle development. Such soft sensors can be based on both physical modeling and data-based modeling. Data-driven modeling is based on building a model purely on captured data which means that no system knowledge is required for the application. At the same time, hyperparameters have a particularly large influence on the quality of the model. These parameters influence the architecture and the training process of the machine learning algorithm. This paper deals with the comparison of different hyperparameter optimization methods for the design of a roll angle estimator based on an artificial neural network. The comparison is drawn based on a pre-generated simulation data set created with ISO standard driving maneuvers. Four different optimization methods are used for the comparison. Random Search and Hyperband are two similar methods based purely on randomness, whereas Bayesian Optimization and the genetic algorithm are knowledge-based methods, i.e., they process information from previous iterations. The objective function for all optimization methods consists of the root mean square error of the training process and the reference data generated in the simulation. To guarantee a meaningful result, k-fold cross-validation is integrated for the training process. Finally, all methods are applied to the predefined parameter space. It is shown that the knowledge-based methods lead to better results. In particular, the Genetic Algorithm leads to promising solutions in this application.

## 1. Introduction

The range of vehicle dynamics control systems and advanced driver assistance systems (ADAS) in current vehicles has already reached a significant level and will continue to grow rapidly [1]. These systems can both support the driver by providing safety, comfort, and efficiency while driving and also allow OEMs to add individual DNA to their vehicles in this way. These functions demand information that the vehicle perceives from the environment. This is mostly done by sensors installed in the vehicle; however, sometimes it is not possible to measure the required signal, or the measurement is very expensive. Instead of physical hardware, mathematical models can be used to observe the required signals. In earlier years, these models were based on physical relationships using the given information to calculate the required value [2,3,4].

Novel approaches use data-driven models, such as Machine Learning (ML) algorithms, to estimate quantities that are difficult to measure, for instance, the sideslip angle or the roll angle. These methods offer the advantage that no system knowledge is required to set up a suitable model; however, the steps to create a model are not trivial. Many engineers proceed without a clearly recognizable strategy leaving much potential unused. This includes the adjustment of external model parameters, such as the architecture of the model or the settings of the optimizer during model training, e.g., the learning rate or the number of neurons. These parameters are called hyperparameters and are not known a priori, so it is the task of the data engineer to find suitable values [5]. A variety of optimization methods can be used for this purpose. This can be a simple grid search or a complex optimization method such as Bayesian Optimization and often a simple optimization is used without considering the advantages of alternatives. Other approaches often promise better results. Comparing state estimators in automotive applications, trial and error, Grid Search or Random Search approaches seem to be commonly used. These approaches promise a simple implementation and first insight into the feasibility [6,7,8,9].

In this paper, optimization methods of different complexity are compared. A basic structure for all the hyperparameter optimizations is created which enables the comparison of four methods. As an application example of a software sensor, this paper discusses a roll angle estimator based on an artificial neural network.

## 2. State of the Art

### 2.1. State Estimation

State estimation in vehicle dynamics is a methodology used when there is no sensor available to directly measure the required state. Mostly, these quantities are needed to detect dangerous driving situations and then to restore a safe driving state with the help of active assistance systems. Direct control of these states can also increase comfort and represent the vehicle manufacturer’s DNA in the vehicle’s dynamic behavior. These measured quantities can be, for example, the roll angle, the float angle, or the self-steering gradient. In this publication, the roll angle is considered in more detail.

**Figure 1:**
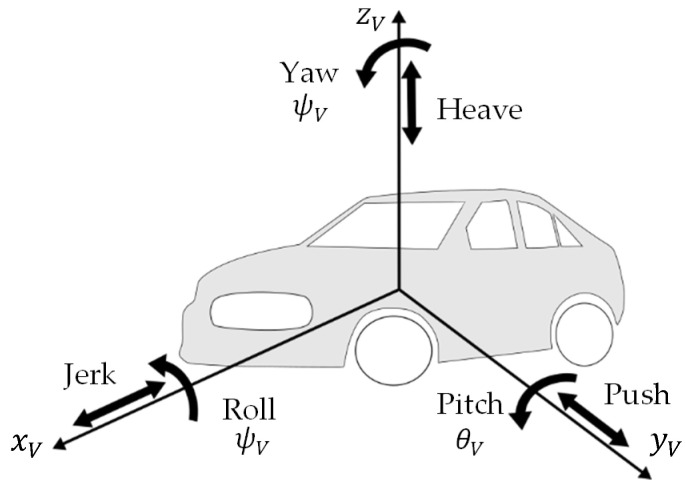
The six rigid body degrees of freedom of a vehicle.

To track the driving states of a vehicle, its spatial motion must be specified. Figure 1 shows the spatial motion of a car, using the body-fixed coordinate system $K_{V}$. The Center of Mass of the body is the origin of the coordinate System $\;K_{V}$.

A vehicle structure modeled as a rigid body has six degrees of freedom that specify its position in space. The roll angle describes the rotation around the longitudinal axis of the vehicle. To estimate this angle a non-linear vehicle model based on the model described in [10] is used, which is:

(1) $$\left(\left(h_{S}-h_{w}\right)m_{a}a_{y}-2A\dot{\varphi }\right)\cos\varphi +\left(\left(h_{S}-h_{w}\right)m_{a}g-2B\right)\sin\varphi -2C\sin^{-1}\left(D\sin\varphi \right)—2E\sin^{-1}\left(F\sin\varphi \right)=J_{xx}\ddot{\varphi },$$

with

(2) $$A=\left(l_{D,v}^{2}d_{D,v}+s_{D,h}^{2}d_{D,h}\right),$$

(3) $$B=\left(l_{F,v}^{2}c_{F,v}+s_{F,h}^{2}c_{F,h}\right),$$

(4) $$C=\frac{c_{St,v}s_{St,v}}{b_{St,v}},$$

(5) $$D=\frac{l_{St,v}}{2b_{St,v}},$$

(6) $$E=\frac{c_{St,h}s_{St,h}}{b_{St,h}}\;\mathrm{and}$$

(7) $$F=\frac{l_{St,h}}{2b_{St,h}}$$

**Figure 2:**
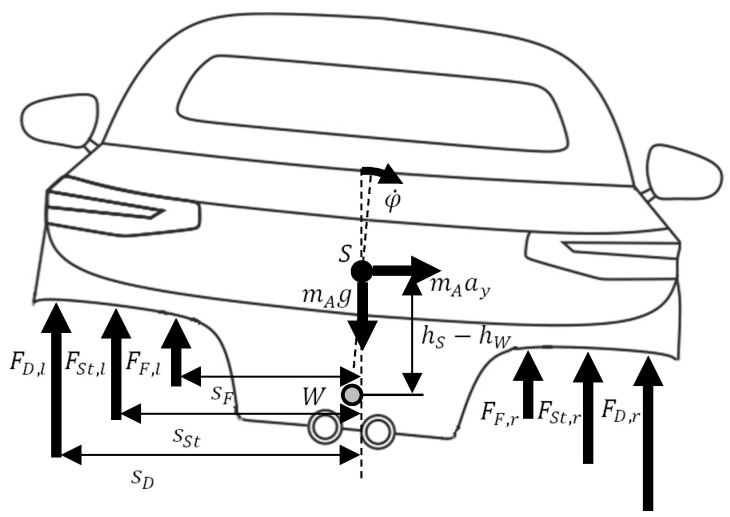
Description of the construction of the rolling dynamics.

Here, φ denotes the roll angle, $J_{xx}$ the moment of inertia around the x-axis, $m_{a}$ the mass of the chassis, $a_{y}$ the lateral acceleration, $c_{S,i}$ is the spring stiffness, $d_{i}$ the attenuation factor and $c_{St,i}$ the stiffness of the stabilizer. The variables $\;l_{St,i},$ and $b_{St,i}$ represent the stabilizer length and the lever arm length of the stabilizer. The values of $\;s_{S,i}$,$\;s_{D,i}$, and $s_{St,i}$ describe the distances between the acting forces and the symmetrical plane of the vehicle body. Figure 2 shows schematically the derivation of the roll dynamics.

Previous works have dealt with the prediction of the roll angle, using physical models for estimation. Thus, Ryu et al. [11] used a physical vehicle dynamic model in combination with an observer to estimate the roll and the bank angle. Based on GPS and inertial navigation system sensors, the roll angle is estimated using a disturbance observer.

Rajamani et al. [12] present algorithms for estimating the roll angle and the height of the center of gravity. The algorithms studied include a sensor fusion algorithm that uses a low-frequency tilt angle sensor and a gyroscope, and a dynamic observer that uses only a lateral accelerometer and a gyroscope. All estimation models are based on physical relationships that are represented in dynamic models.

### 2.2. Machine Learning in Dynamic State Estimation

In addition to model-based methods, machine learning is also finding its way into dynamic state estimation. These methods do not rely on knowledge about the physics of the system. The methods rely on models being trained with empirical data. To do this, a test vehicle must be equipped with a sensor unit to measure the value to be estimated. After training a model with this data, the hardware sensor can be replaced with the so-called software sensor. Different types of machine learning models have been used in the past and this paper presents the use of artificial neural networks.

Sasaki and Nishimaki trained a feedforward net that was used to estimate the side-slip angle. The neural network consisted of three fully connected layers, which were trained using the back-propagation algorithm. The architecture of the model was created by empirical values, which means that no hyperparameter optimization was performed. The selection of hyperparameters was based on previous experience and estimations. Whether the selection of the hyperparameters was optimal cannot be guaranteed [13].

In the paper by Graeber et al. a recurrent neural network was used to estimate the side-slip angle. This was a network that consisted mainly of gated recurrent units. Furthermore, a physical model was connected beforehand, which worked in the style of predictor–corrector models. This publication did not further define how the final architecture was found, i.e., it does not mention if hyperparameter optimization was performed [8].

In the work of Blume et al. and Sieberg et al. recurrent neural networks were used as the basis for estimating the roll angle. The models used in these works were gated recurrent units and long short-term memory cells, which were adapted by hyperparameter optimization [14,15].

Hyperparameters are values that influence the training process and the quality of a prediction. Finding the right network architecture and defining the correct training parameters is a non-trivial task. Therefore, the use of a mathematical optimization technique is mandatory, although so far it is not clear which method yields good results or what the trade-off between the effort and result is for each method.

### 2.3. Hyperparameter Optimization

Two different optimization processes exist when building data-based models. On the one hand, there is the optimization of the model’s internal parameters, which is also referred to as the training process. On the other hand, the so-called hyperparameter optimization, in which external parameters are adjusted that have an influence on the model training or the model architecture. Finding the optimal hyperparameter set can significantly affect the performance of the ML model, which is not a trivial problem and it especially cannot be solved a priori. Often this task is handled using trial and error, which is astonishing since the use of mathematical optimization methods is much more effective.

These methods differ in their complexity and thus in their approach. For example, there are exhaustion methods such as the grid search, which systematically lists all the possible candidate solutions and checks which of the possible candidates best solves the problem. Since testing of all the possible combinations is not practically feasible, the search space is often discretized, and only selected combinations are tested. As this procedure can lead to neglecting part of the search space, it is often modified to randomly generate candidates in the entire search space that are tested for optimality. This procedure is called the Random Search (see Section 3.1). Other methods use the information gained from previous evaluations to select new candidates in a meaningful way, such as Bayesian Optimization (see Section 3.3) or genetic algorithms (see Section 3.4). Yu and Zhu give a general overview of a variety of methods for hyperparameter optimization [16]; however, it is not clear from this comparison which methods are particularly well suited for the development of software sensors.

## 3. Optimization Methods

### 3.1. Random Search

**Figure 3:**
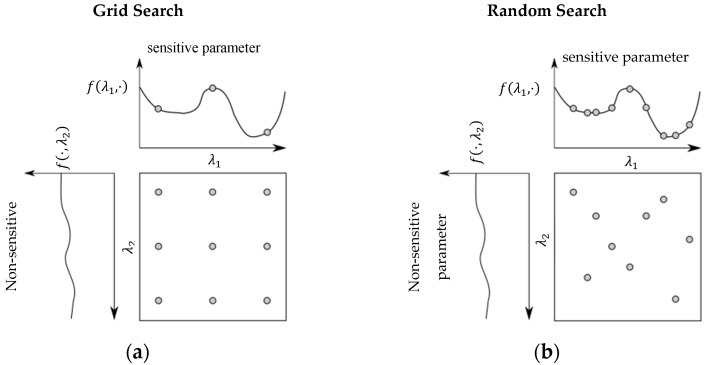
Comparison of Grid Search (**a**) and Random Search (**b**) for minimizing a function with two parameters.

The Random Search (RS) was first introduced by Rastagan in 1963 [17]. RS is an extension of the Grid Search (GS) where a predefined number of parameter sets are randomly selected on the entire search space. The advantage compared to the GS is that the parameter space is much better covered since the individual parameters are less often evaluated on the same values. This is especially the case when one or more parameters are more important than others, as shown in Figure 3. The left axis is the graph of the cost function for the different values of a non-sensitive hyperparameter. As can be seen, the cost function is approximately constant for all values, and a variation of this hyperparameter, therefore, leads to no or only a minimal improvement of the network.

Above, the cost function for a sensitive hyperparameter is shown. Obviously, the value of this hyperparameter has a large influence on the cost function and thus also on the subsequent performance of the network. While only three different values of the important hyperparameter are tested in the grid search, significantly more values are tested in the random search (with the same total number of nine hyperparameter configurations). The probability of finding a local minimum or a better configuration than with the grid search is therefore significantly higher with the random search, but not guaranteed.

Comparing the number of evaluations of GS and RS, we see that with a budget of B function evaluations, a GS only considers B/n parameter settings for each parameter, whereas RS can evaluate up to B different settings for each parameter.

### 3.2. Hyperband

The Hyperband algorithm of Li et al. is a version of the Random Search accelerated by adaptive resource allocation and early-stopping [18]. Here, hyperparameter optimization is formulated as a purely exploratory, non-stochastic, infinite-arm bandit problem. Before starting the optimization, resources are reserved and allocated to randomly selected configurations. Unlike with Random Search, Hyperband does not train all configurations to the last epoch, but only those configurations that show promise.

Hyperband is an extension of the Successive Halving algorithm introduced by Jamieson and Talwalker in 2015 for hyperparameter optimization [19]. For the Successive Halving algorithm, the optimization problem is defined as a non-stochastic best-arm identification problem where each arm corresponds to a fixed hyperparameter setting. Pulling an arm corresponds to a fixed number of training iterations, and the loss corresponds to an intermedia loss on a holdout set. The approach assumes that not all hyperparameter settings need to be trained to convergence, but only the promising ones. An initially defined budget B is uniformly divided among n different hyperparameter configurations, the performances of all configurations are evaluated, and the worst half is removed. This is repeated until only one configuration remains.

In advance, it is unclear whether it be best to choose many configurations (large n) and thus a short training time or few configurations (small n) but a longer training time. The Hyperband algorithm tries to circumvent the problem “n vs. B/n” by testing several possible values for the number of configurations n given a fixed max. number of overall runs B. This approach consists of two loops. The inner loop, called the bracket, involves the Successive Halving algorithm with fixed values for n and r, where r is the minimum resource number allocated to all configurations before discarding some of them. The outer loop iterates over different values for n and r. Each loop iteration results in a different “n vs. B/n” ratio.

Hyperband requires two input values *R* and η. Here, R is the maximum resource that can be provided to a configuration and *η* is the ratio of configurations removed at each pass of the Successive Halving algorithm. These two values result in the number:

(8) $$s_{\max}=[\log_{\eta }\left(R\right)]$$

of the outer loop passes. For each outer loop iteration, the value:

(9) $$\hat{n}=[\frac{B}{R}\cdot \frac{\eta^{s}}{s+1}]$$

which represents the maximum number of hyperparameter configurations, and:

(10) $$\hat{r}=R\cdot \eta^{-s}$$

which is the maximum number of resources available to a configuration, are calculated. Subsequently, n hyperparameter configurations are then randomly created, which are then used in the subsequent successive halving loop. For the inner loop, which corresponds to one iteration cycle of the successive halving, the values:

(11) $$n_{i}=[\hat{n}\cdot \eta^{-i}],\;\;\;i\in \left\{0,\ldots ,s\right\}$$

and

(12) $$r_{i}=\hat{r}\cdot \eta^{i},\;\;\;i\in \left\{0,\ldots ,s\right\}$$

are calculated. The outer loop iterates s from 0 to $s_{\max}$, where the inner loop iterates from 0 to s.

Hyperband starts with the most aggressive allocation of resources $\;s=s_{\max}$, which divides the number n of configurations to maximize exploration. Each loop pass then reduces the number of configurations by the value *η* until the value  s=0, which corresponds to a Random Search. In the last loop pass, *R* resources are then made available to each configuration.

Table 1 shows an example of a Hyperband run with the resources R=400 and the configuration ratio  η=4. During the first loop with $\;s=s_{\max}=4$, the maximum number of configurations, i.e., 400, is trained for just one epoch before the best fourth is selected and this is then trained for six epochs. This is repeated until only the best configuration is left, and this is trained for the full number of resources. In contrast, in loop  s=0 only an ordinary Random Search is performed, i.e., five randomly selected configurations are trained for 400 epochs. Finally, this results in the configuration with the lowest loss.

### 3.3. Bayesian Optimization

Bayesian Optimization is a state-of-the-art optimization method for global optimization. This approach is widely used particularly for computationally intensive black-box models, which includes hyperparameter optimization.

In the mid-1970s, Mockus conducted research in global optimization and conceptualized the optimization of a function as the realization of a stochastic function. These optimization techniques were Bayesian methods, based on minimizing the expected deviation from the extremum [20].

In contrast to Random Search and Hyperband, Bayesian Optimization uses the information obtained from previous evaluations. With the help of the evaluation results, a so-called surrogate model is created, and an acquisition function is used to decide which point should be evaluated next. Surrogate models are often used in optimization problems for which the evaluation of the objective function is either very computationally intensive or impossible [21].

The surrogate is adjusted in each iteration with respect to all previous evaluations of the objective function made so far. Then, the acquisition function, using the predictive distribution of the probabilistic model, evaluates the utility of the different candidate points, weighing exploration against exploitation. This procedure is repeated until a sufficiently good hyperparameter configuration is found or a pre-determined budget is reached. The budget can correspond, for example, to a maximum number of samples or a maximum optimization duration. The advantage of using a surrogate model is that the evaluation of this model is significantly less computationally intensive than the direct evaluation of the objective function and can therefore be optimized more efficiently [22,23,24].

Bayesian Optimization belongs to the Sequential Model-based Optimization methods (SMBO), since it does not optimize the objective function directly, but evaluates the observation history of a probabilistic surrogate model. SMBO algorithms differ mainly in the acquisition function used and in the underlying probabilistic surrogate model.

The method used in this publication is based on Bergstra et al. [22]. There, the criterion, known as expected improvement (EI), is used for optimization because it is intuitive and has been proven in many previous works. Expected improvement is the expectation under some object function $f:\chi \to {\mathbb{R}}^{N}\;$ that f will exceed (negatively) some threshold $\;y^{*}:$

(13) $${\mathrm{EI}}_{y^{*}}\left(x\right)=\int_{-\infty }^{\infty }\max\left(y^{*}-y,0\right)p_{M}\left(y|x\right)dy$$

For the approximation of  f, the Tree-structured Parzen Estimator (TPE) is used as a surrogate model in this contribution. It models p(y|x) by transforming that generative process, replacing the distributions of the configuration with non-parametric densities. To simplify the optimization of EI, the parameterization of p(y|x) as  p(y)p(x|y) in the TPE algorithm was chosen, such that:

(14) $$EI_{y^{*}}\left(x\right)=\int_{-\infty }^{y^{*}}\left(y^{*}-y\right)\frac{p\left(x|y\right)p\left(y\right)}{p\left(x\right)}dy$$

holds. During each iteration, the algorithm passes back the candidate x with the largest EI [22].

### 3.4. Genetic Algorithm

The term “Genetic Algorithm” was introduced in the 1960s by John H. Holland at the University of Michigan, USA. The method we know today was first described in his paper ‘*Adaptation in Natural and Artificial Systems*’. The principle of a Genetic Algorithm of John H. Holland [25], presented in this chapter is taken from the book by Gerdes, Klawonn and Kruse [26]. Structures and operators of simple canonical genetic algorithms are considered in more detail.

The underlying set is called the search space *S* in the following and contains all possible solutions to the optimization problem under investigation. An objective function (or evaluation function) f:S→ℝ is defined on S, which assigns a value $f\left(c_{i}\right)$ to each individual or chromosome $c_{i}$ of the search space S as a measure of its quality. The optimization problem is to find an individual $c_{opt}$ from the search space S that has a minimum (or maximum) score (optimum). The genetic information of an individual is stored in the vector:

(15) $$c_{i}={\left[\begin{array}{cccc} g_{i,1} & g_{i,2} & \cdots & g_{i,m} \end{array}\right]}^{T}.$$

The individuals have the length m, where m corresponds to the number of entries within an individual. These entries are called genes. The value of these genes is called an allele. The chromosomes that evolved and changed during the search run are stored in a population of:

(16) $$P\left(G\right)=\left[\begin{array}{c} c_{1}\\ c_{2}\\ \vdots \\ c_{n} \end{array}\right]=\left[\begin{array}{c} \left[\begin{array}{cccc} g_{1,1} & g_{1,2} & \cdots & g_{1,m} \end{array}\right]\\ \left[\begin{array}{cccc} g_{2,1} & g_{2,2} & \cdots & g_{2,m} \end{array}\right]\\ \vdots \\ \left[\begin{array}{cccc} g_{n,1} & g_{n,2} & \cdots & g_{n,m} \end{array}\right] \end{array}\right]$$

at time G, where G is called generation. This population P(G) is a set of cardinalities  n, i.e., each population of generation G contains n individuals, each of which in turn possesses m genes. For each population P(G) and each individual $c_{i};\;i=1,\ldots \;,n$, the probabilities $p_{C}$ and $p_{M}$ are specified, which indicate whether selected chromosomes cross at time g and whether they mutate, respectively. Here, $p_{C}$ indicates the probability for recombination (crossover) and $p_{M}$ the probability for mutation.

The start population P(0) is a set of cardinalities n and is usually randomly generated. If desired, however, a subset $P_{Start,Fixed}$ of the start population P(0) can be specified, so that only the set $P_{Start,Random}$ with:

(17) $$\left|P_{Start,Random}\right|=n-\left|P_{Start,Fixed}\right|$$

to be generated with random individuals.

However, usually n random chromosomes $c_{i}$ are loaded into the starting population P(0) to minimize the probability of premature convergence (local optimum). For each gene, an upper and a lower bound are defined to ensure the admissibility of these randomly generated individuals. Then, a fitness value is assigned to each individual using the fitness function:

(18) $$F\left(c_{i}\right)=\frac{f\left(c_{i}\right)}{\sum_{j=1}^{n}f\left(c_{i}\right)\;},$$

where $f\left(c_{i}\right)$ is the objective function value of the individual $c_{i}$.

If:

(19) $$\overset{n}{\underset{j=1}{\sum }}f\left(c_{i}\right)=0$$

is valid, then:

(20) $$F\left(c_{i}\right)=\frac{1}{n},\;\;\;\;\forall \;i=1,\ldots ,\;n$$

Is set. In particular, for the fitness function F:

(21) $$\overset{n}{\underset{j=1}{\sum }}F\left(c_{i}\right)=1$$

and

(22) $$0\leq \;F\left(c_{i}\right)\leq 1,\;\;i=1,\ldots ,n$$

is true.

The fitness $F\left(c_{i}\right)$ stands for the survival probability of the individual $\;c_{i}$. The higher the function value $\;F\left(c_{i}\right)$, the higher the fitness of $c_{i}$ and thus the probability that its genes will be transferred to the next generation. Three different operators select or create new individuals for the new generation. On the one hand, so-called elite individuals are selected, which are carried over unchanged into the new generation. These are mainly individuals that have a high fitness value.

On the other hand, another possibility to generate new individuals for the following generation is recombination (crossover). In this process, successful individuals are combined so that new individuals are created that are based on the successful individuals. For the combination of the individuals, different guidelines can be used, whereby not only the strategy but also the data type of the genes is crucial.

To prevent the optimization algorithm from converging locally, since elite and crossover individuals do not generate new information, the so-called mutation operator is used. This operator randomly changes selected genes in selected individuals. Depending on the data type, these changes can be, for example, random values or a permutation of that of the complete individual.

## 4. Data Set

The data used to analyze the different HPOs is based on a data set generated from predefined standard driving maneuvers. The driving maneuvers are based on ISO standards, with characteristic values being adapted. Table 2 shows the different maneuvers, the parameters, and the variations. The road slope is varied for all drives. Other parameters vary depending on the maneuver. For example, the radius can be varied in addition to the direction for the circular maneuvers. For deceleration maneuvers, the maximum deceleration is adjusted, whereas, for acceleration maneuvers, the maximum speed is adjusted. In addition to the maximum and minimum value ranges, the step size is also given so that a complete catalog of relevant test drives can be created from the table.

The maneuver catalog created in this way includes seven driving maneuvers and eight parameters with multiple possible values which result in approx. 4400 different drives. The data is generated with the use of a simulation model in IPG CarMaker.

Since most of the HPOs are computationally very intensive, only a small fraction, 2.5% of the database, meaning 110 driving maneuvers, were selected for hyperparameter optimization. The selection was random, but it was ensured that each driving maneuver was selected at least once. 

**Figure 4:**
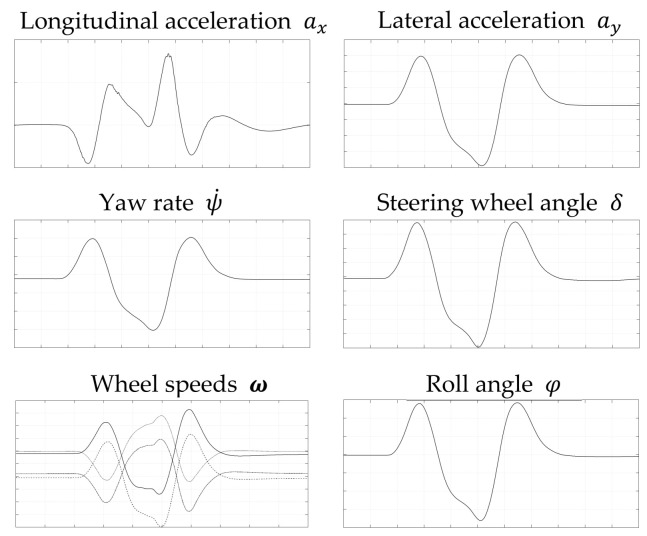
Driving data for the double lane change maneuver.

Figure 4 shows exemplarily the data for a double lane change maneuver. The longitudinal acceleration $a_{x}$, the lateral acceleration $a_{y}$, the yaw rate $\dot{\psi }$, the steering wheel angle δ, the wheel speeds $\omega =\left[\omega_{FL},\omega_{FR},\omega_{RL},\omega_{RR}\right]$ and the roll angle φ are depicted.

## 5. Comparison of HPO

HPO algorithms are compared using the data presented in chapter 4. Thereby the optimization methods described in chapter 3 are analyzed. In all methods, there were two different types of hyperparameters, which were external model parameters. Both types have influence on the architecture of the model or on the optimization process of the internal parameters, i.e., on the training process. On the one hand, there were fixed parameters that were identical for all HPOs, and on the other hand, there were variable parameters that could be adjusted during the optimization process. Table 3 shows the fixed hyperparameters. Besides the standard settings for machine learning algorithms like the division of the data set into the training data (57%), validation data (10%) and test data (33%) as well as the use of the MSE loss, there were also parameters that had already been fixed by empirical values like the use of the optimizer NAdam or the standardization of the data. The limit of 25 epochs was set due to the time required for the training.

Furthermore, there were the hyperparameters that would be adjusted during optimization. These variable hyperparameters are shown in Table 4. Depending on the optimization method, the parameters were changed in different step sizes. Random Search, Hyperband, and the first generation of the Genetic Algorithm created a random parameter set and thus needed the step size. The Bayesian Optimization and the Genetic Algorithm created their parameter combination by calculating new parameter sets from old ones. Thus, the search space was identical to the parameter space, with the RS and Hyperband restricted to discretization by the step size.

The hyperparameters are of three different types, namely integers, real numbers, and categories. The lookback, the number of layers, the number of cells, and the batch size are integers, where the batch size has a step size of 100 and the other three are varied with a step size of 1. The sequence lookback is the number of time steps used recursively to predict the current state. The number of layers is the number of recurrent layers the estimator would have. Since the output layer of the estimator is a fully connected layer, the complete system had (number of layers +1) layer. Due to the architecture of the long short-term memory (LSTM) cell and the gated recurrent unit (GRU), the number of cells is the number of neurons that the gates would have [27,28]. The recurrent layer type is a categorical hyperparameter and decides if the neural estimator will have LSTM cells or GRUs. The number of inputs is also a categorical input, where you can choose from the six different input signals. ${\hat{a}}_{y}$ is a modified form of the lateral acceleration, which can be calculated by:

(23) $${\hat{a}}_{y}=a_{y}+v\cdot \psi .$$

The learning rate, the regularization and dropout values are real numbers in the range between 0 and 0.5, where 0 means that there is no regularization or dropout. The recurrent regularization/dropout is applied to the recurrent connections inside the recurrent layer whereas the normal regularization is applied to the input connections. The batch size and the learning rate are parameters that influence the optimization process. The batch size defines the number of samples that would be propagated through the network before the weights are updated. The learning rate is the step size of the optimization method that updates the weights of the neural network.

**Figure 5:**
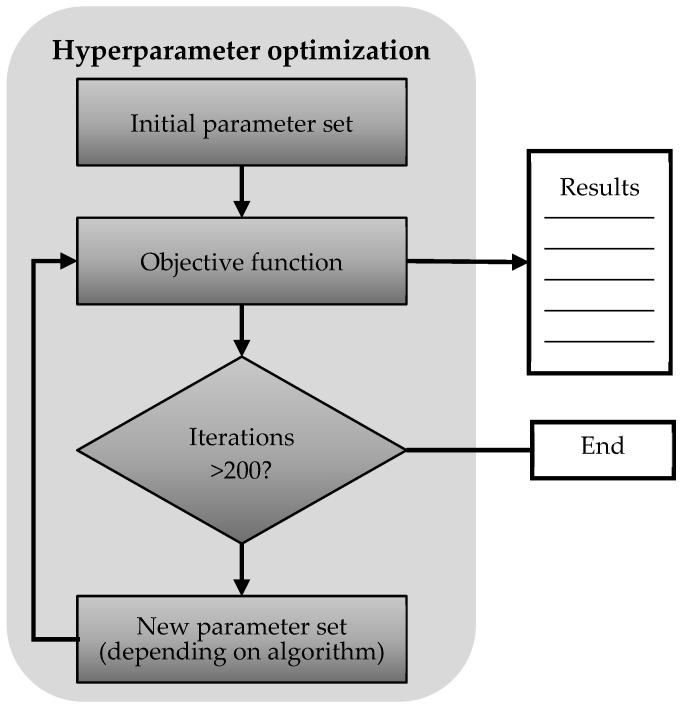
Schematic flow of a general hyperparameter optimization process.

Figure 5 shows the schematic flow of a general hyperparameter optimization. All methods used here use this general principle and differ only in the determination of the new hyperparameter set. The RS and HB only generate random parameters that are not based on previous knowledge, whereas the GA and BO use additional information to generate new parameter sets (see Section 3). The initial and the newly generated parameter sets are evaluated by an object function, i.e., for each element in the parameter set an artificial neural network is generated and subsequently used to determine a predefined performance. The calculated performance for each element in the set is finally stored in a database.

**Figure 6:**
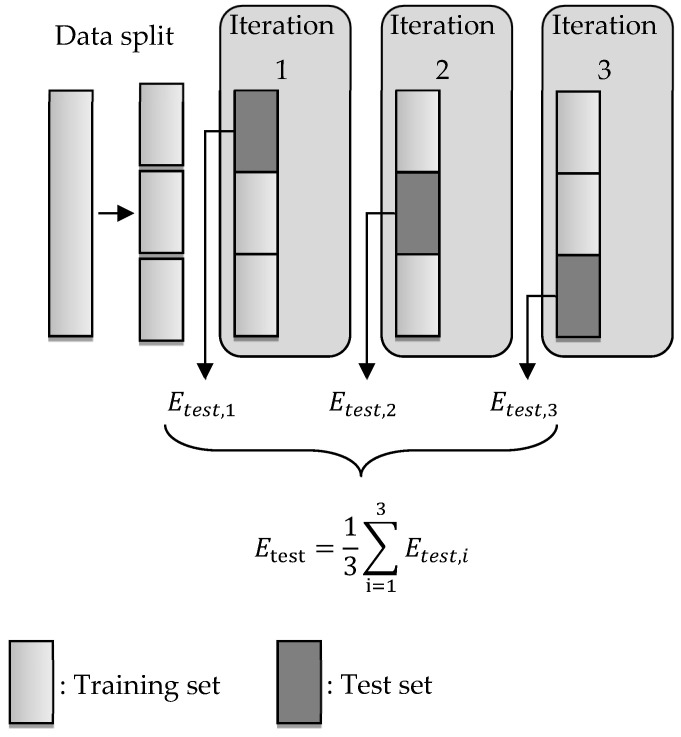
A k-fold cross-validation with k=3.

The hyperparameter optimization uses an objective function that performs a cross-validation to calculate the accuracy of the neural network. This means that the data is divided into k subsets and then each of these k subsets is used as a test data set. Thus, k runs are performed where the kth subset is the test data set, and the other (k-1) subsets are used as the training and validation data set. Figure 6 shows the 3-fold cross-validation that is used for this contribution. Firstly, the shuffled data set is split into three parts. Subsequently, three iterations are performed, where each subset is used once as a test data set. The other two subsets are again subdivided into 85% training data and 15% validation data. For each iteration, the root mean squared error (RMSE) is calculated as the objective function:

(24) $$E_{test,i}\left(\varphi_{pred},\tilde{\varphi }\right)=\sqrt{\frac{1}{n}\overset{n}{\underset{j=1}{\sum }}{\left(\varphi_{j,pred}-{\tilde{\varphi }}_{j}\right)}^{2}},$$

where $\varphi_{pred}=\left[\varphi_{1,pred},\ldots ,\varphi_{n,pred}\right]$ is the prediction of the neural network and $\tilde{\varphi }=\left[{\tilde{\varphi }}_{1},\ldots ,{\tilde{\varphi }}_{n}\right]$ is the reference of the test data set.

In general, the hyperparameter optimization attempts to solve the optimization problem:

(25) $$\underset{W}{\min}E_{test},$$

such that the artificial neural network maps the reference data as accurately as possible, where $E_{test}$ is an arbitrary objective function and W is a matrix containing all weights of the neural network. Both the search space and the objective function are identical for all subsequent HPO methods, so the methods differ only in their criteria for selecting the next hyperparameter configuration to be evaluated. To ensure a fair comparison, each HPO method is also assigned approximately the same budget of 200 iterations. Here, one iteration corresponds to the evaluation of a single hyperparameter configuration on the objective function.

### 5.1. Random Search

**Figure 7:**
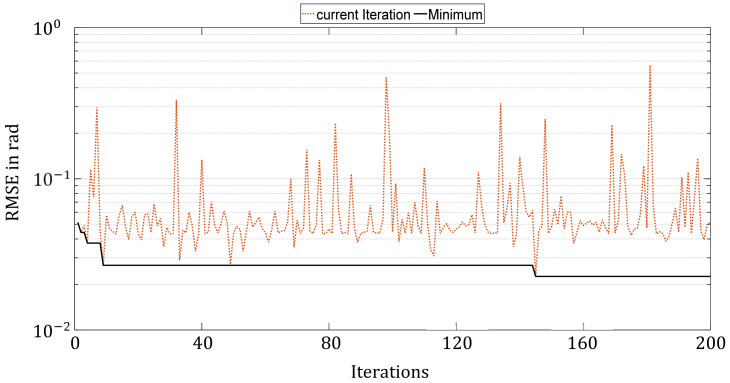
Progress of hyperparameter optimization with Random Search over 200 iterations.

For the RS, hyperparameter sets are generated randomly in the entire search space and successively evaluated using the cost function. The parameters are uniformly distributed over the entire parameter space, which is discretized in terms of step sizes in Table 4. A total of 200 parameter sets were generated and evaluated. Figure 7 shows the progress of the hyperparameter optimization by the Random Search. The RMSE of the hyperparameter configuration evaluated in the current iteration is drawn in dotted orange. The best RMSE found up to the current iteration is represented by the solid black line. The y-axis is on a logarithmic scale.

The figure clearly shows how small the overall improvements are over the entire 200 iterations and that the quality of hyperparameter configurations is subject to major variation. This is since the Random Search approach selects only random parameter sets without taking previous results into account. Nevertheless, this method can coincidentally encounter a good configuration.

### 5.2. Hyperband

Due to the nature of the Hyperband algorithm, not all hyperparameter configurations are trained over the full number of epochs, which in this case was 25 epochs. To ensure a fair comparison of the different HPO methods, several runs were performed for the HP.

Table 5 shows an HP run with R=25, the maximum possible number of epochs to train a configuration, and η=3, the input that controls the fraction of configurations that are discarded in each round of successive halving. From the table, it can now be calculated that one HP run corresponded to approximately 560 training epochs. Since all other HPO methods trained 200 sets for 25 epochs, this resulted in an absolute budget of 5000 epochs for these methods. Nine HP runs must therefore be performed to compare the methods. This corresponds to 5040 epochs.

**Figure 8:**
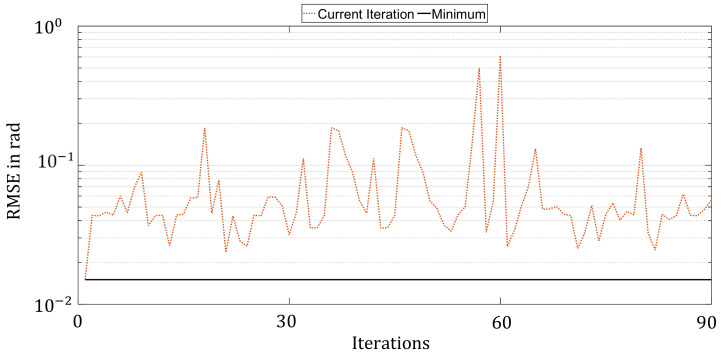
Progress of hyperparameter optimization with Hyperband over 90 iterations, corresponding to 9 HP evaluations.

Comparing the number of generated hyperparameter configurations of HP with the number of the other HPOs shows that significantly more configurations can be considered. In the nine runs, 387 different sets were trained, whereas only 90 configurations were trained over the complete 25 epochs. Thus, Figure 8 shows only 90 iterations.

Looking at the optimization process of the HP algorithm, it is noticeable that HP found an exceptionally good parameter configuration in the first iteration. As mentioned above, this can always happen by chance, although this becomes increasingly improbable as the parameter space becomes larger; however, most of the evaluated parameter settings are significantly inferior. No configuration comes into this quality range or even gets better.

### 5.3. Bayesian Optimization

**Figure 9:**
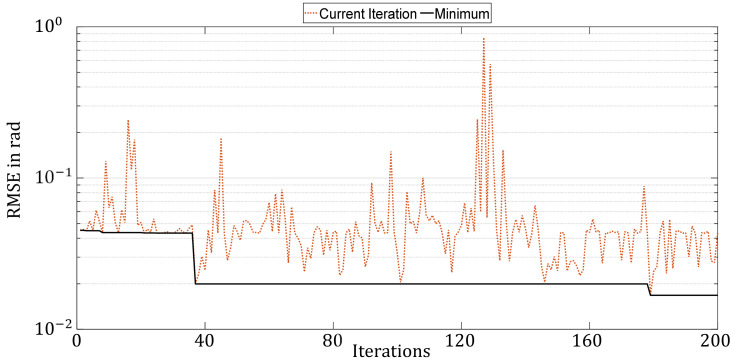
Progress of hyperparameter optimization with Bayesian Optimization over 200 iterations.

To minimize the HPO using Bayesian Optimization, an initial set must first be generated. For this purpose, 20 random parameter sets were created and evaluated. Then, using the surrogate model and the acquisition function as described above, another 180 parameter configurations were evaluated. Each new selection of parameters is based on the information generated in the previous results. That is, a surrogate model is created and minimized using the acquisition function. The result obtained is the configuration that is evaluated in the new iteration. Figure 9 depicts the development of the function evaluations of the optimization process.

It can be seen that the RMSE tended to decrease with a continuous iteration number, although upward swings could be seen again and again. This can be observed in particular between the iterations 120 and 140. In contrast to the Random Search and Hyperband approaches, however, a clear trend can be seen.

### 5.4. Genetic Algorithm

The initial population for the Genetic Algorithm was randomly generated. For this purpose, 40 individuals were generated in the entire search space using the step sizes from Table 4. After evaluating the initial population, 40 individuals were generated for each additional generation using the genetic operators shown in Table 6.

**Figure 10:**
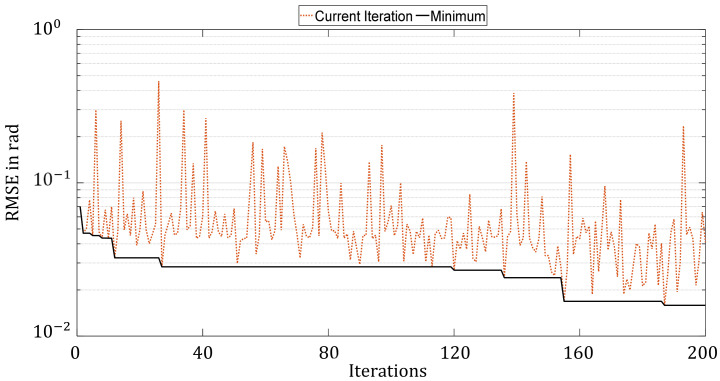
Progress of hyperparameter optimization with the Genetic Algorithm over 5 generations with 40 individuals each, corresponding to 200 training iterations.

From the previous population and the individuals generated by the genetic operators, 40 more individuals were selected for the new generation. Figure 10 shows that an improvement of the configurations occurs in almost every generation. Just as with the Bayesian Optimization, a decreasing trend can be seen; however, significant oscillations can be seen in the evaluations, which can be explained by the mutations. In this way, local convergence can be prevented, and new areas of the parameter space can be explored.

## 6. Results

The results of all hyperparameter optimization methods are shown in Table 7. Comparing the RMSE of the four methods, it is noticeable that the Bayesian Optimization and the Genetic Algorithm give results that differ only slightly in their structure. Whereas the five best results of the Random Search and Hyperband are very widely scattered over the parameter space. When dropout and regularization are considered, it is noticeable that the best results are obtained when neither dropout nor regularization is used; however, recurrent regularization and dropout are selected. Therefore, the weights within the layers are influenced, but the weights between the layers are not. This is due to the “knowledge” of the two algorithms, BO and GA, whereas the RS and HB just randomly select parameter sets. Of course, this can also lead to randomly selecting the global optimum. This can be seen in the best parameter setting of Hyperband, which found a good configuration in the first iteration step but could not improve afterward. It was even the case that the other configurations were significantly worse than this outlier. Similarly, it can be seen that the LSTM cells often gave better results than the GRU. The learning rate should also be selected as rather low. Only the size of the meshes differs significantly. There were relatively small networks (hyperband) with only 1 layer and 35 neurons as well as large networks (GA) with 4 layers and 37 neurons each. At this point, it must be stated that due to the increased number of weights in the large networks, an increase in the number of epochs could lead to better results. Additionally, in the lookback, all results below 0.02 rad had values between 2 and 10, and the lookback leads to a significantly larger net structure, because the recurved nets are unrolled for training.

**Figure 11:**
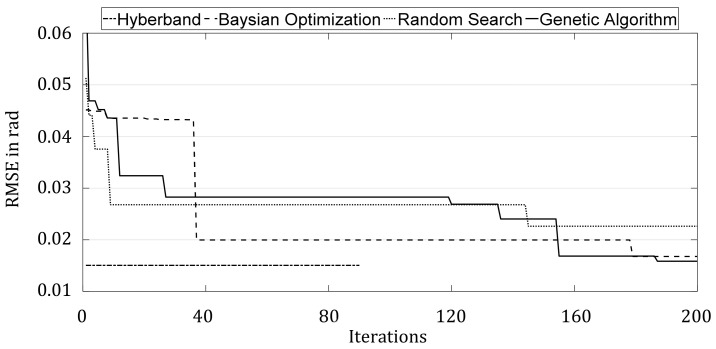
Comparison of best HPO results plotted over all iterations.

Figure 11 compares the optimization histories of the four HPO methods. Since Hyperband does not train all the selected configurations over the full number of epochs, only 90 iterations are shown in the figure. HP found the best configuration directly in the first iteration and did not improve in the further course. Whereas the other methods improved successively. While the Bayesian Optimization and Random Search took a few large steps to improve, the Genetic optimizer improved more frequently in small steps in each generation, but it is clear from the BO and GA that there was a steady improvement. It is reasonable to assume that increasing the number of iterations would have led to further improvement in the results.

## 7. Conclusions

In this paper, four hyperparameter optimization methods were compared using an artificial neural network-based roll angle estimator as an example. Two random-based methods, namely, Random Search and Hyperband, and two knowledge-based methods, namely, Bayesian Optimization and the Genetic Algorithm, were compared. All four algorithms were given the same data and an identical budget of resources to optimize the hyperparameter of the software sensor. The results showed that the Random Search and the Hyperband Algorithms, which are both based exclusively on random selections, do not have a continuous improvement during the optimization process. Instead, after finding a good parameter configuration, it is likely that a parameter setting will be selected that does not improve because it has no relation to the previous selection. Nevertheless, these algorithms can lead to satisfactory results and if one has a small search space, these methods can also converge quickly. On the other hand, if we compare the Bayesian Optimization or the Genetic Algorithm with the two previously mentioned, it is clear that the knowledge that goes into the generation of new parameter configurations leads to a continuous improvement of the parameter selection. In summary, knowledge-based methods are preferable to random-based methods for the optimization of a large number of hyperparameters, and thus for a large search space and a strongly nonlinear problem. The increased effort in implementing such methods often leads to better results and is, therefore, a worthwhile investment. Of course, purely random-based methods can also lead to good results, but this is less likely due to the size of the search space. Among other reasons, this is because there are normally no hints on how to narrow down the hyperparameter spaces.

## Figures and Tables

**Table 1:** Hyperband algorithm with *R* = 400 and *η* = 4.

** i **	** s=4 **		** s=3 **		** s=2 **		** s=1 **		** s=0 **	
	$n_{i}$	$r_{i}$	$n_{i}$	$r_{i}$	$n_{i}$	$r_{i}$	$n_{i}$	$r_{i}$	$n_{i}$	$r_{i}$
**0**	256	1	80	6	27	25	10	100	5	400
**1**	64	6	20	25	6	100	2	400		
**2**	16	25	5	100	1	400				
**3**	4	100	1	400						
**4**	1	400								

**Table 2:** Driving maneuver catalog that is used to generate training, test, and validation data.

Maneuver	Parameter	Values	Step Size
Braking from Steady-state circle	Direction	CW/CCW ^1^	-
	Deceleration	2.5 to 12 m/s²	0.5 m/s²
	Slope	−8° to 8°	1°
Braking test	Velocity	30 to 120 km/h	10 km/h
	Slope	−8° to 8°	1°
Double lane change	Direction	CW/CCW	-
	Velocity	30 to 120 km/h	10 km/h
	Slope	−8° to 8°	1°
Power-off reaction from Steady-state circle	Direction	CW/CCW	-
	Max. lat. Acc.	4 to 12 m/s²	0.5 m/s²
	Slope	−8° to 8°	1°
Steering-pulse	Direction	CW/CCW	-
	Velocity	30 to 120 km/h	10 km/h
	Steer Angle	−180° to 180°	60°
	Slope	−8° to 8°	1°
Sinus steering	Frequency	0.2 Hz to 2 Hz	0.45 Hz
	Velocity	30 to 120 km/h	10 km/h
	Slope	−8° to 8°	1°
Steady-state circular test	Direction	CW/CCW	-
	Radius	40 m/100 m	-
	Velocity	30 to 120 km/h	20 km/h
	Slope	−8° to 8°	1°

^1^ CW: clockwise; CCW: counterclockwise.

**Table 3:** Fixed hyperparameters.

Name of Hyperparameter	Value
Optimizer	Nadam
Loss	MSE
Number of Epochs	25 ^1^
Scaler	Standardization
Validation Data Split	10%
Test Data Split	33%

^1^ All HPOs run 25 epochs per training run except for Hyperband. The algorithm does not train all configurations over the complete number of epochs (see Section 3.2).

**Table 4:** Variable hyperparameter and their parameter space.

Name of Hyperparameter	Minimum	Maximum	Step Size
Sequence Lookback	1	10	1
Inputs	$\left\{a_{x}\right\},\left\{a_{y}\right\},\left\{{\hat{a}}_{y}\right\}$ {δ},{ψ},{ω}	$\left\{\begin{array}{c} a_{x},a_{y},{\hat{a}}_{y},\\ \delta ,\psi ,\omega \end{array}\right\}$	*-*
Number of Layers	1	6	1
Number of Cells	1	50	1
Recurrent Layer Type	LSTM, GRU	-	-
L2-Regularization value	0	0.5	0.001
Recurrent L2- Regularization value	0	0.5	0.001
Dropout value	0	0.5	0.001
Recurrent Dropout value	0	0.5	0.001
Batch Size	100	1000	100
Learning Rate	0.001	0.3	0.001

**Table 5:** Hyperband configuration for *R* = 25 and *η* = 3.

** i **	** s=4 **		** s=3 **		** s=2 **		** s=1 **		** s=0 **	
	$n_{i}$	$r_{i}$	$n_{i}$	$r_{i}$	$n_{i}$	$r_{i}$	$n_{i}$	$r_{i}$	$n_{i}$	$r_{i}$
**0**	16	1	10	3	7	6	5	12	5	25
**1**	8	3	5	6	3	12	2	25		
**2**	4	6	2	12	1	25				
**3**	2	12	1	25						
**4**	1	25								

**Table 6:** Genetic parameters.

Genetic Operators	
Crossover	Uniform crossover
Mutation	Uniform mutation
Selection	Tournament selection

**Table 7:** The 5 best parameter sets found by Random Search, Hyperband, Bayesian Optimization and Genetic Algorithm.

RMSE in rad	Look-back	Inputs	Layers	Neurons	Type	L2-Reg	Recurrent L2	Dropout	Recurrent Dropout	Batch Size	Learning Rate
Random Search											
0.022620	4	$\left\{a_{x},a_{y},\dot{\psi }\right\}$	3	25	LSTM	0	0.47	0.151	0.39	500	0.05
0.026779	7	$\left\{a_{y},\delta ,\dot{\psi }\right\}$	0	7	LSTM	0.058	0.345	0	0.052	400	0.054
0.027185	8	$\left\{a_{y},\delta ,\dot{\psi },\omega \right\}$	3	28	LSTM	0	0	0.404	0.193	900	0.013
0.028898	8	$\left\{a_{x},{\hat{a}}_{y},v,\dot{\psi }\right\}$	0	35	GRU	0.392	0.196	0.24	0.087	900	0.015
0.030892	9	$\left\{a_{x},a_{y},{\hat{a}}_{y},\delta \right\}$	0	15	LSTM	0.062	0.103	0.405	0.006	900	0.073
Hyperband											
0.01505	10	$\left\{\begin{array}{c} a_{x},a_{y},{\hat{a}}_{y},\\ v,\delta ,\dot{\psi } \end{array}\right\}$	1	35	LSTM	0	0.439	0	0.443	600	0.009
0.02362	1	$\left\{a_{x},a_{y},{\hat{a}}_{y},\omega \right\}$	1	12	LSTM	0	0.02	0.352	0.383	300	0.001
0.02452	3	$\left\{\begin{array}{c} {\hat{a}}_{y},v,\delta ,\\ \dot{\psi },\omega \end{array}\right\}$	0	30	LSTM	0	0.174	0.05	0.277	700	0.02
0.02526	10	$\left\{\begin{array}{c} a_{x},a_{y},{\hat{a}}_{y},\\ \delta ,\omega \end{array}\right\}$	2	43	GRU	0.052	0.003	0.254	0.45	600	0.013
0.02605	3	$\left\{\begin{array}{c} a_{x},a_{y},v,\\ \delta ,\dot{\psi } \end{array}\right\}$	3	9	LSTM	0	0.08	0.354	0.245	700	0.005
Bayesian Optimization											
0.016771	8	$\left\{a_{y},{\hat{a}}_{y},\delta \right\}$	4	18	LSTM	0	0.395	0	0.417	486	0.007
0.019957	9	$\left\{a_{x},{\hat{a}}_{y},v\right\}$	3	12	LSTM	0	0.34	0	0.005	270	0.032
0.020468	10	$\left\{a_{y},{\hat{a}}_{y},\delta \right\}$	3	8	GRU	0	0	0	0	481	0.013
0.020470	9	$\left\{a_{x},v,\delta \right\}$	5	7	LSTM	0	0.319	0	0.49	476	0.014
0.022709	10	$\left\{a_{x},\delta ,\dot{\psi }\right\}$	2	11	LSTM	0	0.358	0	0.401	686	0.04
Genetic Algorithm											
0.015857	4	$\left\{a_{x},a_{y},\dot{\psi }\right\}$	4	37	LSTM	0	0.410	0	0	100	0.006
0.016851	7	$\left\{a_{x},{\hat{a}}_{y},v,\dot{\psi }\right\}$	2	49	LSTM	0	0	0	0.317	800	0.012
0.017035	10	$\left\{a_{x},a_{y},{\hat{a}}_{y},\dot{\psi }\right\}$	0	44	LSTM	0.489	0	0	0	100	0.047
0.017035	2	$\left\{a_{x},\delta ,\dot{\psi }\right\}$	1	10	LSTM	0.407	0.237	0	0.465	200	0.112
0.018027	8	$\left\{a_{x},\delta ,\dot{\psi }\right\}$	1	28	LSTM	0	0.408	0	0.465	700	0.247
